# Impact of Supplementing Phytobiotics as a Substitute for Antibiotics in Broiler Chicken Feed on Growth Performance, Nutrient Digestibility, and Biochemical Parameters

**DOI:** 10.3390/vetsci9120672

**Published:** 2022-12-03

**Authors:** Anastasiya S. Zaikina, Nikolai P. Buryakov, Maria A. Buryakova, Artem Yu. Zagarin, Artem A. Razhev, Dmitrii E. Aleshin

**Affiliations:** Department of Feeding Animals, Institute of Animal Science and Biology, Russian State Agrarian University—Moscow Timiryazev Agricultural Academy, 127434 Moscow, Russia

**Keywords:** poultry, antibiotic, phytobiotic, growth performance, digestibility, biochemical parameters

## Abstract

**Simple Summary:**

The rapid growth in antibiotic synthesis and the discovery of new classes of antibiotics have led to their wide availability. Antibiotics have begun to be used not only in medicine but also in the agricultural sector. The widespread, uncontrolled use of antibiotics in industrial poultry farming has contributed to the growth of resistant types of pathogenic and opportunistic environmental microorganisms. In addition, the vigorous introduction of antibiotics contributes to the risk of the contamination of livestock products, which are then used in human nutrition with antibiotic residues present. The consumption of poultry products contaminated with antibiotic residues due to antibiotic resistance can be lethal to humans. Therefore, we hypothesize that feed additives based on plant extracts, which can be used as alternatives to antibiotics, can affect the productivity, health status, and quality of poultry products. Our results show that the use of phytobiotics in the diet of broilers had promising effects on the digestibility of feed nutrients and the dynamics of the growth of the live body weight of broiler chickens.

**Abstract:**

To determine the level of application and the effectiveness of the use of plant feed additives from sweet chestnut wood extract (*Castanea Sativa Mill*) in the diet of Cobb-500 cross broiler chickens, four groups were formed via the balanced groups method. The chickens in the experimental groups were supplemented with sweet chestnut wood extract in the main diet rather than a feed antibiotic at an amount of 500 g per ton of compound starter feed and 250 g per ton of grower and finisher in the second experimental group; 650 g per ton of compound starter feed and 325 g per ton of grower and finisher in the third experimental group; and 800 g per ton of compound starter feed and 400 g per ton of grower and finisher in the fourth experimental group. Supplementation with phytobiotics led to an increase in the digestibility of the dry matter in the second and third groups compared to the first experimental group. Furthermore, broiler chickens supplemented with a medium dose of phytobiotics revealed a significant difference in both crude protein and fiber when compared to the second experimental group (91.95% and 12.11% vs. 88.98% and 10.07%, respectively). The preslaughter weight of the birds in the phytobiotics supplemented groups was higher than in those fed with the lowest dosage of phytobiotic by 5.47%, and the difference was significant. There were no significant differences in terms of the blood biochemical parameters between the groups. In summary, the inclusion of plant feed additives from sweet chestnut wood extract as a substitute for an antibacterial drug in the diet of broiler chickens did not deteriorate the blood biochemical parameters and improved the intensity of the nutrient digestion process. As a result, it enhanced the quality indicators of the broiler carcass during the entire growth period.

## 1. Introduction

In modern agricultural science, more advanced methods are needed in the development of technological processes for the preparation of feed and feed additives. To feed farm animals, the use of biologically active elements, sorbents, and natural stimulants can help us to obtain environmentally friendly and high-quality agricultural foodstuffs [1,2,3,4,5,6,7,8,9]. Therefore, to achieve the genetic potential of an animal and preserve its health, the search for new feed additives that improve the quality of feed and increase its value is relevant to scientists in the field of animal nutrition all over the world [10,11,12,13,14,15]. In industrial poultry farming, infectious diseases are treated with antibiotics; however, unfortunately, they affect not only pathogenic microorganisms but also the normal intestinal microflora, since the destructive effect of the antibiotics is not selective. Antibiotics in the poultry sector are used not only for the prevention and treatment of respiratory and gastrointestinal manifestations but also as growth stimulants [16,17,18,19]. The haphazard and continued use of antibiotics in large doses, especially those with a wide spectrum type of action, has led to the selection of pathogenic microflora that are resistant to these drugs in the environment. As a result, new, more powerful drugs are created, and more resistant bacteria types appear [19,20,21].

Recently, the efforts of many specialists in the field of poultry farming have been aimed at finding new physiological and environmental approaches, as well as means of activating the body defenses of broiler chickens, to increase their safety and productive traits. Among these biotechnological methods is the use of phytobiotics, which allow for the suppression of microbial growth and the stimulation of metabolic processes and productivity due to the presence of a mixture of herbs and plant extracts with flavoring, aromatic, therapeutic, and prophylactic properties. Many studies have been devoted to improving the technology of poultry production, as it is the most dynamic branch of the agro–industrial complex. One of the approaches to successfully developing poultry farming is the development and creation of feed additives and growth stimulants of natural origin [22,23]. The development of new effective, natural, environmentally friendly herbal preparations (phytobiotics) as an alternative to antibiotics is one of the crucial tasks of recent biological science [24,25].

In recent years, a large number of data have been accumulated that indicate the feasibility of substituting traditional feed antibiotics with phytobiotic feed additives and their positive impact on the quality of final products [1,4,5,11,13,20,22,26,27,28,29,30,31,32,33]. An analysis of the literature demonstrated the prospects of using phytobiotics in poultry farming due to their pronounced positive effect on productivity, nonspecific resistance, and the physiological state of poultry. Plant extracts and purified derivatives show positive results in poultry feeding by increasing feed efficiency and maximizing economic efficiency. Tannins are compounds of plant origin that are successfully used as additives in poultry feed to fight diseases and improve animal productivity. The effective use of any of these extracts as feed additives ensures the production of a stable quality product in an amount sufficient to meet the actual needs of the population. Chestnut (hydrolysable) and quebracho (condensed) tannins are the most affordable commercial products that meet these needs [30]. Although tannins can have a beneficial effect on nutrient digestibility and, subsequently, on poultry productivity when included in their diets, their main mechanism of action is not sufficiently known to fully explain their effects on poultry [30,31].

Some authors propose that a low concentration of tannins can improve the palatability of feed and increase the productivity of animals with a single-chamber stomach by stimulating feed consumption [32]. Others suggest that the stimulation of the digestive secretion is the main mode of action [33]. However, tannins’ antimicrobial properties seem to be their most important mechanism of action, especially in young animals. In general, similar to antibiotics, compounds of plant origin participate in the modulation of a very complex interaction between the microbiota and the gastrointestinal tract. The resulting release of the host animals from harmful microbial activity and their undesirable products may cause lower costs of immune protection [32,34,35,36,37]. However, the complexity of the interactions and dynamics of the intestinal microbiota makes it difficult to quantify such effects. With this in mind, the aim of our research was to study the effect of commercial plant feed additives from sweet chestnut wood extract (phytobiotic) as a replacement for antibiotics in the diet of Cobb 500 cross broiler chickens on the growth parameters, nutrient digestibility, and biochemical parameters of the poultry enterprise in central Russia.

## 2. Materials and Methods

The study was conducted in accordance with the standards and legislation of the Russian Federation and approved by the Ethics Committee of the Russian State Agrarian University–Moscow Agricultural Academy named after K.A. Timiryazev (protocol 2022-5 date 5 February 2022).

### 2.1. Animals and Experimental Design

This study was conducted in the joint-stock company “Verkhnevolzhskaya Poultry Farm” in the Kalininsky district of the Tver region (Russia) in 2019–2022. The objects of research were Cobb-500 cross broiler chickens. The duration of the experiment was 38 days.

The construction of groups of birds for the experiment, as well as the scientific and economic experiments, were carried out in accordance with the recommendations of the Federal State Budgetary Scientific Institution of the Federal Scientific Center of the All-Russian Scientific Research and Technological Institute of Poultry Farming of the Russian Academy of Sciences [38]. Four groups were formed via the balanced groups method (control and experimental groups 1, 2, and 3) (Table 1). Conditioned Cobb-500 cross chickens were divided by weight at one day of age. The chickens were raised on the floor with a litter of sawdust. All day-old chickens were examined by a full-time veterinarian from the poultry farm before the start of the experiment, and no diseases or sharp deviations from the normal clinical state of health were noted.

The broiler chickens from each group were kept in separate poultry houses, which were single-room poultry houses with a floor-mounted poultry growing system. The conditions of feeding and keeping in all groups were identical and corresponded to the technological parameters of keeping Cobb-500 cross broiler chickens. The broilers were fed with full-fledged compound feeds that comply with the recommendations for the cross. The compound feed was manufactured by Smolinsky Combine of Bakeries. The inclusion of a phytobiotic preparation was carried out by introducing it into the compound feed. The broiler chickens in the control group received a main diet supplemented with the feed antibiotic Flavomycin^®^ (FLA). The chickens in the experimental groups were supplemented with SCWE in their main diet instead of a feed antibiotic at an amount of: compound starter feed—500 g per ton, grower and finisher—250 g per ton for experimental group 2; compound starter feed—650 g per ton, grower and finisher—325 g per ton for experimental group 3; and compound starter feed—800 g per ton, grower and finisher—400 g per ton for experimental group 4. Five days before the slaughter of the broilers, the antibiotic and phytobiotic were removed from the compound finisher feed.

### 2.2. Feed, Animal Feeding, and Dietary Supplement

The diet of the animals was balanced in accordance with the recommendations for feeding Cobb 500 cross broiler chickens. With the help of the Feed Optima program (v. 2020.8.17251), they were balanced to meet the needs of poultry in terms of energy, proteins, lipids, carbohydrates, vitamins, and minerals in different growing periods. Feed and drinking water were given freely throughout the experiment with the help of hopper feeders and nipple drinkers. Before conducting the research, all the poultry in the control and experimental groups underwent a mandatory vaccination program against infectious diseases according to the scheme used at the farm.

The chemical composition, ingredients, energy value, and content of minerals and vitamins in the daily diet are presented in Table 2 and Table 3.

Flavomycin^®^ was used as an antimicrobial drug—the antibiotic presents as a granular fermentation product of *Streptomyces ghanaensis* (manufacturer Huvepharma^®^, Sofia, Bulgaria). The sweet chestnut wood extract plant feed additive (Farmatan BCO (Butitan)) was made by Tanin Sevnica d.d. (Slovenia). It consisted of a complex of micro-capsulated tannins with butyrate and calcium lactate and the essential oils of cinnamon, oregano, and chili pepper. The main active ingredient in this feed additive is an extract from sweet chestnut wood (*Castanea Sativa Mill*), which is obtained by water extraction without the use of chemicals. The extract contains several active substances (flavonoids, organic acids and their salts, saponins, mono- and polysaccharides, essential oils, micro- and macroelements, etc.), the most abundant of which are hydrolysable ellagitannins [35,36,37].

Samples were sent to the Scientific Testing Center in Cherkizovo to measure the chemical composition of the supplement fed to the experimental animals. Feed samples were taken in accordance with ISO 6498:2012 Animal Feeding Stuffs—Guidelines for Sample Preparation [39].

In the samples, the chemical compositions of the raw materials were determined by using AOAC methods [40]:

- Dry matter by drying at a temperature of 100 °C (DM; method 934.01);
- Crude ash (method 942.05);
- Crude protein (CP; method 968.06);
- Essential extract (EE; method 920.39);
- Crude fiber (CF, method Ba 6-84);
- Calcium and phosphorus (ISO 27085:2009) [41].

### 2.3. Analysis of Growth Performance and Quality of Meat

During the research, the following indicators were taken into account [38,42]:

1. The live weight (g) was determined by the control individual weighing of broiler chickens (100 heads from each group) at the age of 7, 14, 21, 28, 32 days, and before slaughter.
2. The absolute gain (A, g) is the increase in live weight over the period of the experiment; it was determined using Equation (1):

(1) $$A=W_{1}-W_{0}$$

where *W*_1_ is the live weight of the broilers at the end of the growing period (final BW, g) and *W*_0_ is the live weight of the broilers at the beginning of the growing period (initial BW, g).

3. Average daily gain (ADG, g) was calculated by weighing the results; it was determined using Equation (2):

(2) $$V_{t}=\frac{W_{2}-W_{1}}{t_{2}-t_{1}}$$

where *W*_2_ is the live weight of broilers at the end of the growing period (g), *W*_1_ is the live weight of the broilers at the beginning of the growing period (g), *t*_2_ is the age of the chicks at the end of the growing period (days), and *t*_1_ is the age of the chicks at the beginning of the growing period (days).

4. Feed cost per 1 kg of live weight gain (kg) was calculated by dividing the amount of feed consumed over the entire period of the experiment by the live weight gain of the broiler chickens during the growing period.
5. Productivity index (points) was calculated using Equation (3):

(3) $$Ip=\frac{LM\times Sp\times 100}{Pv\times Wk}$$

where *Ip* is the productivity index (points), *LM* is the average live weight (kg), *Sp* is the livestock safety (%), *Pv* is the duration of cultivation (days), and *Wk* is the feed cost per 1 kg of gain (kg).

To assess the effect of feeding the studied feed additive on the meat productivity of broiler chickens at the age of 38 days, the chickens were slaughtered, and we then conducted the anatomical cutting of the chicken carcasses according to the method outlined by the Federal State Budgetary Scientific Institution Federal Scientific Center of the All-Russian Scientific Research and Technological Institute of Poultry Farming of the Russian Academy of Sciences (2013). For this purpose, three cockerels from each group were selected that had average indicators of body weight and fatness in the group.

### 2.4. Digestibility of Nutrients

The digestibility and use of nutrients of the diets were established based on the results of balance experiments conducted at the age of 18 days according to the guidelines of the Federal State Budgetary Scientific Institution of the Federal Scientific Center of the All-Russian Scientific Research and Technological Institute of Poultry Farming of the Russian Academy of Sciences (2015). For this experiment, five broilers from each group were selected that were homogeneous in live weight and reflected the average for the group. The birds were kept in special cages with a mesh floor, under which a retractable litter tray was installed. White paper and a plastic film were laid on the pallet. The poultry was distributed over four diets and kept separately in one-story cages, making sure that the chickens were distributed evenly.

In order to compile an average sample, animal feed, remains, and excreta were taken and stored in plastic jars each day. Feed and leftovers were weighed twice a day, once in the morning and one in the evening, during the entire period of the experiment to calculate the consumption and digestibility of the nutrients. The litter was carefully collected from the pallet, the feathers and fluff were removed, and then it was weighed. For preservation, 0.1 N oxalic acid solution was added to the litter at a rate of 1 mL per 50 g of litter. The added amount of acid was taken into account when determining humidity. Before the analysis, the excrement of each chick for three days was combined.

The indicators were taken into account for each individual. During the experiment, feed consumption and the amount of excreted litter were carefully taken into account. At the end of the experiment, the samples were dried in a drying cabinet at 65 °C for several days and crushed for further chemical analysis.

The analysis of the chemical composition of the litter was carried out similarly to the methods presented in Section 2.3. The feed intake was calculated as follows (4):

(4) Feed consumption= offered feed−remaining feed

The digestibility coefficient (DC) of each nutrient in the diet was evaluated using Equation (5):

(5) $$\mathrm{DC}=\frac{\mathrm{intake}\;\mathrm{nutrient}-\mathrm{excreted}\;\mathrm{in}\;\mathrm{feces}}{\mathrm{intake}\;\mathrm{nutrient}}\times 100\%$$

The individual feed consumption for the poultry house was recorded weekly. We consistently measured the total body weight, average daily gain, feed conversion rate, and daily food intake per herd.

### 2.5. Blood Sampling and Analysis

Blood for biochemical examination was taken in the morning from the axillary vein from five broilers from each group aged 36 days into vacuum tubes with a coagulant coagulation activator (Zhejiang Gongdong Medical Technology Co., Ltd., Huangyan, China).

Blood serum was obtained after blood sampling and the centrifugation of blood samples for 15 min, 3000× *g*, and then stored at a temperature from +4 °C to +8 °C to determine the biochemical parameters of the blood.

The blood test was carried out at an independent certified veterinary laboratory (Moscow) using a Beckman Coulter AU 480 device (Beckman Coulter, Inc., Brea, CA, USA). According to the results of the blood test, the concentration of glucose, total protein, albumins, globulins, urea, uric acid, creatinine, cholesterol, triglycerides, calcium, and phosphorus in the blood serum was determined.

### 2.6. Statistical Analysis

The results are expressed as mean ± standard error. All data were subjected to Shapiro–Wilk’s and Levene’s tests to test the normality and homogeneity of the data before running the statistical tests. The data were statistically analyzed using the statistical analysis program SPSS (2017). One-way ANOVA followed by Duncan’s multiple range tests and Tukey’s multiple comparison tests (post hoc test) were used to compare the means of the treated groups, with *p* < 0.05 being considered statistically significant.

## 3. Results

### 3.1. Growth Performance and Meat Qualities of Broiler Chickens

The dynamics of the live body weights of broiler chickens are shown in Table 4.

At the age of 7 days, the live weights of chickens exceeded the control in the third experimental group by 1.0% and in the fourth experimental group by 4.1%, but the live weight of control chickens was higher than in the second experimental group by 3.6%. At the age of 14 days, the live weight of the chickens in the third experimental group was 0.4% higher when compared to the control group. In the fourth experimental group, it was 7.3% higher than in the control group; however, the control chickens outperformed the second experimental group by 4.6%. From the data given in Table 5, it can clearly be seen that the average daily increments had large values in the experimental chickens treated with phytobiotics (SCWE).

At the beginning, the average daily gains in live weight in the third experimental group exceeded those of the control group by 1.0%, and the fourth group outperformed the control group by 5.3%. The greatest difference in the average daily gains can be observed in the period from 14 to 21 days, where the growth rates of chickens in all the experimental groups exceeded those of the control group—by 3.1% in the second experimental group, by 6.9% in the third experimental group, and by 2.7% in the fourth experimental group.

One of the main factors determining the level of profitability of livestock and poultry farming is the safety of the livestock (Table 5). During our study, it was found that the safety of livestock in the third and fourth experimental groups with the introduction of a phytobiotic (SCWE) was 0.1 and 0.4% higher compared to the control group, respectively. However, in the second experimental group, where broiler chickens received the lowest level of phytobiotic, the safety was lower compared to the control and other experimental groups. At the same time, the highest safety of broilers was observed in the fourth experimental group (98.1%), where the maximum amount of SCWE was introduced into the compound feed.

The cost of feed per unit of production is one of the main sources of expenditure and accounts for 60–70% of all the costs involved in livestock production. This indicator has a great impact on the economic efficiency of the grow broilers. When calculating feed consumption per 1 kg of body weight gain (Table 5), it was found that in the broiler chickens of the third and fourth experimental groups, this indicator was at the level of 1.47 and 1.48 kg, which is less than the level of the control group by 3.3 and 2.6%, respectively.

The lowest feed cost per 1 kg of gain was found in the third experimental group of poultry that was receiving 650 g/ton of phytobiotic in the starter feed and 325 g/ton in the grower and finisher feed. One of the important indicators of the efficiency of the use of production facilities and equipment is the yield of broiler meat per unit of production area of the poultry house (1 m^2^), which is calculated by taking into account the final livestock amount and the useful area of the floor.

When feeding the chickens a phytobiotic preparation based on sweet chestnut extracts, an increased in meat yield per 1 m^2^ of floor area can be observed as the level of input of SCWE increased. Thus, the highest yield of meat was obtained in the fourth experimental group—47.3 kg/m^2^, which is 8.5% higher than in the control. When SCWE was used in feeding, the lowest meat yield of 44.7 kg/m^2^ was found in broilers that received 500 g/ton of phytobiotic in the starter feed and 250 g/ton in the grower and finisher feed (2nd experimental group).

In our studies, it was found that when phytobiotic (SCWE) was introduced into the poultry feed, the productivity index in the third and fourth experimental groups was high and amounted to 483.6 and 491.9 units, which is 4.2% and 5.9% higher than in the control group, respectively.

It was found that broiler chickens in the fourth experimental group had the highest live weight, greatest average daily growth and safety, and the lowest feed cost per unit of production. In this group’s feed, antibiotic Flavomycin was replaced with a phytobiotic drug (SCWE) at a concentration of 800 g/ton in the starter feed and 400 g/ton in the grower and finisher feed.

According to the results of the broilers slaughtered at the age of 38 days (Table 6), it was found that the highest index of preslaughter live weight was 2781 g (*p* < 0.05) in the cockerels of the fourth group (SCWE 3), which is 5.47% (*p* < 0.05) higher compared to the third experimental group, which was supplemented with the lowest level of phytobiotic. The weights of uneviscerated chickens from the SCWE 2 and SCWE 3 groups exceeded those of the SCWE1 group by 6.07% (*p* < 0.05) and 9.48% (*p* < 0.05), respectively.

However, in relation to the control group, significant differences were noted in the fourth group (2643.69 g), which was supplemented with the highest level of phytobiotic feed additive. 

There were no significant differences in the mass of the gutted carcass and its yield in relation to the live body weight in the studied groups. The anatomical cutting of carcasses was carried out for the complete assessment of the meat quality of broiler chickens. The pectoral muscles are the most valuable because they have high protein content. The mass of these muscles in the control group was 573.75 g, while in the third and fourth experimental groups, it was 6.10% and 13.51% higher, respectively, with supplementation with a phytobiotic. The mass of the leg muscles, including the mass of the lower leg and thigh, in the control group was 509.8 g, while in the SCWE 3 group, it was higher by 98.61 g.

### 3.2. Intake and Digestibility of Nutrients

Based on the actual consumption of feed and the excreted litter, the coefficients of the digestibility of the nutrients in the feed of poultry in the control and experimental groups were determined. The results are presented in Table 7.

The digestibility coefficient of dry matter in the control group, where FLA was used, was 73.03%. When the compound feed of broilers included SCWE, it led to an increase in the digestibility of dry matter by 2.04% and 1.72% in the second and third groups, respectively. In terms of protein digestibility, the best indicator was in the second experimental group, amounting to 91.95–2.03% higher than in the control group. The digestibility coefficient of crude protein in SCWE 1 was the lowest (88.98%) compared to SCWE 3 broiler chickens. The digestibility of crude fiber in the control group was 11.72%. In SCWE 1, this indicator was the lowest (10.70%), and in SCWE 3 it was the highest (1.47%) (*p* < 0.05) when compared to the first experimental group.

### 3.3. Biochemical Parameters Blood Serum

The serum contents of total protein, albumin, globulin, protein index, urea, uric acid, creatinine, glucose, cholesterol, triglycerides, total calcium, and inorganic phosphorus, as well as the Ca/P ratio, were measured. The results obtained are presented in Table 8.

The glucose concentration in the blood serum of broiler chickens recorded non-significant differences between the experimental groups and ranged from 13.1 to 13.6 mmol/L. With regard to the total protein as a criterion of protein metabolism, the highest value was recorded in the control group. When analyzing the lipid metabolism data, unreliable differences in the concentration of cholesterol and triglycerides between the groups were obtained, although the groups with phytobiotics had the lowest triglyceride content. In general, the introduction of phytobiotics at different levels did not affect changes in the biochemical parameters of the blood. All obtained biochemical indicators for all groups were within the reference values.

## 4. Discussion

### 4.1. Growth Performance and Meat Quality of Broiler Chickens

The inclusion of a phytobiotic in the diet of broiler chickens had a positive effect on the productivity indicators. According to the results obtained after the slaughtering of broilers at 38 days of age, it was found that the highest index of preslaughter live weight was in the cockerels of the fourth group (SCWE 3), which was higher than in the control group (SCWE 1), and the differences were significant. The mass of the gutted chickens from the SCWE 2 and SCWE 3 groups exceeded that of the lowest phytobiotic level group by 6.07% (*p* < 0.05) and 9.48% (*p* < 0.05), respectively, probably due to the fact that plant extracts can affect the peristalsis of the stomach and the process of digestion in the intestine [43]. Similar results were obtained in the work of Gheisar et al. (2015) [44], who reported that the addition of phytobiotics to broiler diets led to a 3.9% increase in live weight.

The effect of phytobiotics on the growth rates of chickens has yet to be determined. Some authors cite data that show minor responses [33,45,46], while others have found a significant impact on productivity [15,47,48,49].

A similar situation was observed in animals according to the mass of half-eaten carcasses. This was likely due to the greater digestibility of nutrients in the experimental groups due to the inclusion of phybiotics, which contributed to a more intensive increase in live weight, especially the half-gutted carcass and leg muscles.

The pectoral muscles are the most valuable because they have a high protein content. The mass of these muscles in the control group was 573.75 g, while the figures for the third and fourth experimental groups were 6.10% and 13.51% higher, respectively. The mass of the leg muscles, including the mass of the lower leg and thigh, in the control group was 509.8 g, and in the group with phytobiotic (SCWE 3) this indicator was higher by 98.61 g. Similar results were obtained by Aljumaah et al. (2020) [50], who found that feeding a mixture of mint oils to broiler chickens for 35 days affected the output of pectoral and leg muscles (*p* < 0.05), and the birds that received the oils had a higher output of pectoral muscles compared to the birds that received the antibiotic Avilamycin.

The data on the growth performance and meat quality of broiler chickens are not reliable (*p* > 0.05); however, they are consistent with the results obtained by Zdunczyk et al. (2010) [51], who found a significant increase in the pectoral muscle mass in broilers receiving a diet with Sangrovit (a phytobiotic).

Some authors claim that there is still no accurate understanding of the mechanism of influence of phytobiotics on poultry productivity, growth indicators, and animal health. The use of various phytobiotic feed additives is due to the composition and activity of the components, which depend on a certain amount of input. Changing the dose of these drugs can lead to an ambiguous response in animals. The content and composition of biologically active substances are primarily determined by the type of plant, the agricultural techniques involved in its cultivation, the processing methods used, the harvest time, plant diversity, and interactions with other feed ingredients [52].

### 4.2. Intake and Digestibility of Nutrients

Feed additives are a broad group of feed components that can affect biochemical processes and can cause a desired reaction in animals to digestion processes, as well as the digestibility of nutrients, growth, and metabolism [2,53].

Many substances found in nature have a wide range of growth-stimulating, immunostimulant, or antimicrobial activity. Phytogenic extracts are a relatively new type of additive compared to antimicrobial drugs (antibiotics), pH regulators, antioxidants, and enzymes [49,54,55]. Despite the fact that these supplements have demonstrated antioxidant and antimicrobial efficacy, as well as the effectiveness of immune stimulation in in vitro experiments, in vivo data are still very limited. A limited number of studies compare phytogenic feed additives with acidifiers [53,56].

In our study, we noted lower feed intake in the groups receiving the phytobiotic compared with the group receiving the antibacterial drug (*p* > 0.05). All the experimental groups, with the exception of those supplemented with the minimum level of phytobiotic feed additive, showed lower indicators for the digestibility of nutrients such as dry matter, crude protein, essential extract, and crude fiber, all of which were below the level of the control group. This can probably primarily be attributed to the lower dosage of the phytobiotic feed additive, which was insufficient to stimulate the formation of enzymes and mucus in the intestinal lumen. Some studies have shown [15,56,57] that the use of plant feed additives contributes to an increase in the synthesis and activity of enzymes and increases the production of bile acids, and the effect of essential oils on increasing the activity of trypsin and amylase in broilers has also been established [33,46].

Previously, researchers have shown that the inclusion of various plant extracts improves the digestion and absorption of nutrients in the intestine [55]. Some authors [58], on the contrary, have argued that the inclusion of plant extracts in the diets of chickens leads to an increase in the secretion of intestinal mucus, thereby increasing the digestibility of nutrients.

### 4.3. Biochemical Parameters Blood Serum

The results of our present research showed that feeding a phytobiotic based on sweet chestnut extract with calcium butyrate did not significantly affect the biochemical blood parameters of chickens during the growth period. It has been suggested that various components of plant origin may have hypocholesterolemic effects in broiler chickens [33,54]. In the current study, we did not observe hypocholesterolemic effects caused by the use of a phytogenic feed additive; however, lower values of triglycerides and cholesterol were noted compared to the control. When analyzing the lipid metabolism data, non-significant differences in the concentration of cholesterol and triglycerides were obtained between the groups, and the phytobiotic-supplemented groups had the lowest levels.

Similar results were obtained by Aljumaah et al. (2020) [50] when using phytobiotics instead of the antibiotic Avilamycin. The effect of phytobiotics on the blood parameters of broilers at the age of 35 days did not change the total protein, albumin, globulin, or cholesterol (*p* > 0.01) amounts in this study. However, there was a significant decrease in the level of glucose and triglycerides in the blood. When conducting research on the use of phytobiotic additives based on micro-capsulated essential oils, Moharreria et al. (2022) [58] also noted a decrease in aminotransferases in the blood of broiler chickens infected with *Salmonella* enteritidis.

When analyzing the biochemistry of blood, unreliable differences in the concentration of cholesterol and triglycerides were obtained between the groups, although the phytobiotic groups had the lowest triglyceride levels. Generally, the introduction of phytobiotics at different concentrations did not reveal variations in blood biochemical parameters. Moreover, all the obtained biochemical parameters in all groups were within the reference values.

Based on the results of one study, an *Satureja hortensis* L. extract significantly reduced the plasma glucose levels, AST, and ALT in broilers exposed to heat stress (*p* < 0.05) [20,59].

In conclusion, it is possible to use food additives with different compositions based on plant compounds instead of an antibiotic to more effectively maintain growth indicators and improve the meat quality of broilers. In general, the introduction of phytobiotics at different concentrations did not cause harmful changes in the blood biochemical parameters.

## 5. Conclusions

Based on our results, the supplementation of plant feed with additives from sweet chestnut wood extract (Castanea Sativa Mill) as a substitute for an antibacterial drug in the diet of Cobb 500 cross broiler chickens did not harmfully affect the biochemical parameters of blood throughout the rearing period. At the same time, it improved the intensity of the nutrient digestibility process, as well as the quality indicators of the poultry carcass. Thus, we recommend the inclusion of the plant feed additive sweet chestnut wood extract (Castanea Sativa Mill) at a dose of 400 g per 1 ton of compound feed as the most appropriate dose in the diet of Cobb 500 cross broiler chickens.

## Figures and Tables

**Table 1:** Feeding scheme and research design.

Groups	Poultry Quantity	Features of Feeding Broiler Chickens
FLA	28,891	Basic diet (BD) + antibiotic Flavomycin^®^ (FLA), presented as a granular fermentation product of *Streptomyces ghanaensis*
SCWE 1	28,965	BD + plant feed additives from sweet chestnut wood extract (SCWE) at an amount of: poultry aged up to 10 days—500 g per ton of compound feed; poultry aged from 11 to 34 days or more—250 g per ton
SCWE 2	28,853	BD + SCWE at an amount of: poultry aged up to 10 days—650 g per ton of compound feed; poultry aged from 11 to 34 days or more—325 g per ton
SCWE 3	30,929	BD + SCWE at an amount of: poultry aged up to 10 days—800 g per ton compound feed; poultry aged from 11 to 34 days or more—400 g per ton

**Table 2:** Feed content in the rations of broiler chickens (%).

Ingredients	Age of Poultry (Days)			
	0–10 (Starter)	11–21 (Grower)	22–33 (Finisher 1)	34–38 (Finisher 2)
Whole wheat grain	–	10.00	10.00	–
Wheat grain crushed	36.07	26.80	34.18	47.53
Corn grain crushed	17.28	20.00	17.00	14.80
Full-fat soybean	5.00	8.00	10.00	13.00
Soybean meal	32.51	22.41	11.82	6.67
Sunflower meal	–	2.00	5.00	7.00
Fish meal	2.68	–	–	–
Meat and bone meal	–	3.00	4.00	4.04
Sunflower oil	2.76	4.24	4.84	4.44
Lysine monochlorohydrate	0.32	0.45	0.53	0.42
DL-methionine	0.39	0.41	0.36	0.29
L-threonine	0.15	0.21	0.19	0.17
Sodium chloride	0.24	0.16	0.14	0.19
Sodium bicarbonate	0.21	0.25	0.22	0.15
Monocalcium phosphate	0.68	0.58	0.34	0.05
Limestone powder	0.71	0.49	0.38	0.25
Vitamin–trace mineral premixes	1.00	1.00	1.00	1.00
Antibiotic Flavomycin^®^ (FLA) *	150	150	150	–

* Only in control group. Unit: grams per 1 ton as part of the premix.

**Table 3:** Nutritional value of the rations of broiler chickens.

Indicator	Age of Poultry (Days)			
	0–10 (Starter)	11–21 (Grower)	22–33 (Finisher 1)	34–38 (Finisher 2)
	Nutritional Value			
Metabolic energy (kcal/100 g)	303	316	322	322
Crude fiber (%)	3.57	3.58	3.73	4.00
Crude protein (%)	22.7	20.6	19.0	18.7
Lysine (%)	1.43	1.34	1.25	1.13
Methionine (%)	0.71	0.69	0.63	0.56
Methionine + cystine (%)	1.06	1.02	0.94	0.87
Threonine (%)	0.96	0.92	0.83	0.80
Tryptophan (%)	0.28	0.24	0.21	0.21
Assimilable lysine (%)	1.29	1.20	1.12	1.00
Assimilable methionine (%)	0.68	0.65	0.59	0.52
Assimilable methionine + cystine (%)	0.96	0.91	0.83	0.75
Assimilable threonine (%)	0.83	0.79	0.70	0.67
Assimilable tryptophan (%)	0.23	0.20	0.17	0.17
Essential extract (%)	5.24	7.35	8.30	8.30
Linoleic acid (%)	2.71	3.80	4.30	4.29
Calcium (%)	1.00	0.96	0.93	0.85
Phosphorus (%)	0.77	0.74	0.71	0.66
Phosphorus assimilable (%)	0.50	0.48	0.45	0.40
Potassium (%)	0.94	0.83	0.71	0.68
Sodium (%)	0.20	0.20	0.20	0.20
Chlorine (%)	0.28	0.26	0.27	0.28
Dietary Electrolyte Balance (meq/100 g)	25.2	22.8	19.5	18.6

**Table 4:** Dynamics of live weight (1 head) of broiler chickens (g).

Age of Poultry	Type and Level of Growth Promoters				*p*-Value
	FLA	SCWE 1	SCWE 2	SCWE 3	
1 day	48.00 ± 0.87	47.70 ± 0.69	48.00 ± 0.77	47.80 ± 0.80	0.99
7 days	193.77 ± 3.38 ^ab^	186.95 ± 3.77 ^b^	196.02 ± 3.78 ^ab^	202.23 ± 5.34 ^a^	0.08
14 days	518.37 ± 6.35 ^bc^	494.33 ± 8.32 ^c^	520.33 ± 6.17 ^b^	555.51 ± 5.42 ^a^	0.001
21 days	1038.37 ± 14.04 ^b^	1030.28 ± 12.94 ^b^	1076.40 ± 11.05 ^ab^	1089.58 ± 12.67 ^a^	0.001
28 days	1690.00 ± 22.91	1680.00 ± 21.98	1688.00 ± 21.17	1708.00 ± 21.06	0.83
35 days	2317.00 ± 31.83 ^ab^	2268.00 ± 41.46 ^b^	2398.00 ± 40.98 ^ab^	2441.00 ± 44.00 ^a^	0.01
38 days	2745 ± 31.17	2712 ± 48.14	2762 ± 36.88	2820 ± 38.30	0.56

Values are expressed as mean ± standard error. FLA—antibiotic Flavomycin^®^, SCWE—plant feed additives from sweet chestnut wood extract. Means denoted within the same row with different superscripts are significant (*p* < 0.05).

**Table 5:** Zootechnical indicators of growing broiler chickens.

Parameters	Type and Level of Growth Promoters			
	FLA	SCWE 1	SCWE 2	SCWE 3
Quantity of poultry (*n*)	28,891	28,965	28,853	30,929
Average live weight (1 day), g	48.0	47.7	48.0	47.8
Average live weight (38 days), g	2745	2712	2762	2820
Average live weight (only ♂, 38 days), g	2494.70 ^a^	–	–	2635.60 ^b^
Average live weight (only ♀, 38 days), g	2340.30	–	–	2344.86
Total daily live weight gain, g	2697	2664.3	2714	2772.2
Average daily live weight gain, g	71.0	70.1	71.4	73.0
Safety of livestock, %	97.7	96.2	97.8	98.1
Feed costs per 1 kg of live weight gain, kg	1.52	1.52	1.47	1.48
Meat production from 1 m^2^ of area, kg/m^2^	43.6	44.7	45.6	47.3
Productivity index, units	464.3	451.73	483.6	491.9

Values are expressed as means. Means within the same row with different superscripts are significantly different (*p* < 0.05). FLA—antibiotic Flavomycin^®^, SCWE—plant feed additives from sweet chestnut wood extract.

**Table 6:** The quality of the carcass of broiler chickens.

Parameters	Type and Level of Growth Promoters			
	FLA	SCWE 1	SCWE 2	SCWE 3
Finish weight (g)	2691.67 ± 22.42 ^ab^	2636.67 ± 38.44 ^b^	2715.00 ± 28.43 ^ab^	2781.00 ± 19.35 ^a^
Uneviscerated poultry (g)	2519.89 ± 27.55 ^bc^	2414.78 ± 26.65 ^c^	2561.40 ± 29.10 ^ab^	2643.69 ± 20.21 ^a^
Semieviscerated poultry (g)	2224.97 ± 25.08 ^bc^	2144.64 ± 15.95 ^c^	2274.98 ± 24.49 ^ab^	2361.65 ± 28.45 ^a^
The weight of the eviscerated carcass (g)	1967.26 ± 95.16	1947.13 ± 59.52	2064.03 ± 37.03	2197.34 ± 19.06
The weight of the pectoral muscles (g)	573.75 ± 11.92	563.90 ± 28.89	608.81 ± 33.52	651.33 ± 63.85
Leg muscles mass weight (g)	509.82 ± 4.14 ^ab^	461.59 ± 7.58 ^b^	538.79 ± 10.49 ^ab^	560.20 ± 35.51 ^a^

Values are expressed as mean ± standard error. Means within the same row with different superscripts are significantly different (*p* < 0.05). FLA—antibiotic Flavomycin^®^, SCWE—plant feed additives from sweet chestnut wood extract. Means denoted within the same row with different superscripts are significant (*p* < 0.05).

**Table 7:** Digestibility of nutrients by broiler chickens aged 18–20 days.

Parameters	Type and Level of Growth Promoters				*p*-Value
	FLA	SCWE 1	SCWE 2	SCWE 3	
Intake feeds (g)	101.22 ± 4.02	98.72 ± 2.70	98.30 ± 2.41	98.94 ± 3.06	0.910
Dry matter (%)	73.03 ± 0.42 ^ab^	72.37 ± 0.48 ^b^	74.41 ± 0.37 ^a^	74.09 ± 0.34 ^a^	0.010
Crude protein (%)	89.92 ± 0.48 ^bc^	88.98 ± 0.46 ^c^	91.95 ± 0.42 ^a^	90.90 ± 0.36 ^ab^	0.001
Essential extract (%)	84.86 ± 0.52	83.68 ± 0.33	85.43 ± 0.52	85.32 ± 0.47	0.065
Crude fiber (%)	11.72 ± 0.26 ^ab^	10.70 ± 0.34 ^b^	12.11 ± 0.39 ^a^	11.91 ± 0.26 ^ab^	0.028
Nitrogen-Free Extractive Substances (%)	81.38 ± 0.59	81.70 ± 0.72	82.37 ± 0.54	81.87 ± 0.66	0.738

Values are expressed as mean ± standard error. Means within the same row with different superscripts are significantly different (*p* < 0.05). FLA—antibiotic Flavomycin^®^, SCWE—plant feed additives from sweet chestnut wood extract. Means denoted within the same row with different superscripts are significant (*p* < 0.05).

**Table 8:** Biochemical parameters of blood serum of broiler chickens (36 days).

Parameters	Units of Measurement	Type and Level of Growth Promoters			
		FLA	SCWE 1	SCWE 2	SCWE 3
Glucose	mmol/L	13.6 ± 0.34	13.6 ± 0.44	13.3 ± 0.36	13.1 ± 0.36
Creatinine	umol/L	22.2 ± 0.74	22.4 ± 1.11	22.8 ± 0.63	22.8 ± 0.58
Total protein	g/L	33.2 ± 1.11	32.7 ± 0.37	33.5 ± 0.87	33.8 ± 0.97
Albumin	g/L	13.0 ± 0.63	12.9 ± 0.62	12.8 ± 0.66	12.8 ± 0.49
Globulin	g/L	20.2 ± 0.58	19.8 ± 0.73	20.7 ± 0.53	21.0 ± 0.55
Urea	mmol/L	0.76 ± 0.040	0.82 ± 0.06	0.75 ± 0.05	0.74 ± 0.040
Uric acid	umol/L	256.8 ± 45.89	273.76 ± 52.17	252.80 ± 44.09	251.0 ± 41.65
Cholesterol	mmol/L	3.46 ± 0.153	3.34 ± 0.190	3.53 ± 0.200	3.52 ± 0.139
Triglycerides	mmol/L	0.92 ± 0.102	0.71 ± 0.080	0.68 ± 0.090	0.65 ± 0.062
Total calcium	mmol/L	2.36 ± 0.216	2.23 ± 0.130	2.54 ± 0.080	2.61 ± 0.043
Inorganic phosphorus	mmol/L	2.87 ± 0.210	2.75 ± 0.190	2.66 ± 0.070	2.58 ± 0.027
Ca/P ratio	–	0.85 ± 0.114	0.80 ± 0.150	0.96 ± 0.050	1.01 ± 0.014

Values are expressed as mean ± standard error. FLA—antibiotic Flavomycin^®^, SCWE—plant feed additives from sweet chestnut wood extract. Ca/P ratio—the ratio of calcium to phosphorus.

## Data Availability

The raw data supporting the conclusions of this article will be made available by the authors without undue reservations. The data presented in this study are available upon request from the corresponding author.
